# CO_2_-assisted fabrication of silica gel adsorbent in honeycomb rotary wheels for air dehumidification

**DOI:** 10.3389/fchem.2022.1038095

**Published:** 2022-10-04

**Authors:** Junjie You, Junbo Qin, Chuanqing Du, Jianhua Fu, Siqing Cheng

**Affiliations:** ^1^ Innovation Centre for Nanomaterials in Energy and Environment (ICNEE), School of Chemical and Environmental Engineering, Wuhan Polytechnic University, Wuhan, Hubei, China; ^2^ Wuhan Coobase Facilities Company Limited, Wuhan, Hubei, China

**Keywords:** honeycomb rotary wheel, dehumidification, CO_2_, silica gel, absorbent

## Abstract

A honeycomb rotary wheel fabricated from sheet adsorbent of silica gel is a competitive drying facility for air dehumidification in modern drying and air conditioning industries due to its large contacting area (3,000 m^2^/m^3^) and the rapid diffusion of the adsorbate compared to silica gel pellets. The delicate preparation procedure of hygroscopic silica gel is paramount for improved adsorption capacity by optimizing the surface area, pore size, and pore volume of silica gel. In this article, silica gel adsorbent in a honeycomb rotary wheel was fabricated by neutralizing the impregnated water glass solution with a modulus of 3.3 on the glass fiber sheet of the honeycomb matrix using CO_2_ at different pressure at room temperature instead of corrosive acids. The as-obtained silica gel absorbent was characterized by XRD, scanning electron microscopy (SEM), specific surface area and pore size analysis, and dynamic vapor/gas sorption analysis. The results showed that the as-obtained silica gel adsorbent is uniform in size and tunable in terms of specific surface area, pore size, pore volume, and adsorption capacity by CO_2_ pressure. The typical silica gel fabricated by CO_2_ of 0.25 MPa with a specific surface area of 764.86 m^2^/g, an average micropore size with a diameter of 2.94 nm, and a pore volume of 0.45 ml/g delivers a saturated adsorption capacity of as high as 287.24 mg/g at RH 50%, which is the best in adsorption performance compared to the previously reported results. This provides a new strategy for environment-friendly manufacturing of silica gel adsorbent in honeycomb rotary wheels for air dehumidification.

## Introduction

Air humidity has always been drawing a worldwide concern both in our daily life and in various conventional (food, cosmetics, papermaking, pharmaceuticals, etc.) and emerging industries (microelectronics, lithium-related batteries, etc.) due to its role with respect to air quality and its requirement for the manufacture, preservation, transportation, and further processing of products (Aristov et al., 2002; Wu et al., 2018). Thus, air dehumidification technology has been attracting extensive interest academically and industrially. The traditional direct dehumidification technologies mainly consist of condensing dehumidification, heating dehumidification, membrane dehumidification, and liquid desiccant dehumidification, in which the relevant environmental issues owing to the utilization of a non-environment-friendly refrigerant, the energy-intensive requirement, high-cost membrane fabrication, etc. impede their further development, especially in today’s ever-increasing climate deterioration (Cha et al., 2007; Hwang et al., 2007; Bhagat et al., 2008; Iswar et al., 2017). In recent years, an adsorptive dehumidification technology by solid regenerable desiccant to selectively remove and separate the moisture from moist air under ambient conditions shows great promise for future air dehumidification (Kabeel, 2007; Bareschino et al., 2015).

Thus far, four types of solid desiccant dehumidifiers as adsorptive dehumidification technology have been proposed, namely, solid packed beds, rotating horizontal beds, multiple vertical beds, and honeycomb rotary wheels, among which only the honeycomb rotary wheels integrate the dehumidifier and desorption processes into one, demonstrating the lower pressure drop and large surface area for heat and mass transfer with respect to the desirable characteristics, such as high space efficiency, continuous adsorption–desorption cycles, high dehumidification, and utilization of low-grade heat sources, such as solar energy and waste heat, for adsorbent regeneration (Chung et al., 2001; Bhagat et al., 2008; Hindersmann, 2019). As a state-of-the-art solid desiccant dehumidification technology, the honeycomb rotary wheel consists of numerous parallel airflow passageway constructed typically with alternating sinusoidal corrugated fibrous substrate sheets and flat fibrous substrate sheets on whose surface a solid desiccant material is impregnated. As such, the widely dispersed desiccants work based on the famous Kelvin formula which shows the vapor pressure difference between the air and the desiccant to attract the moisture from the air until its vapor pressure is in equilibrium with the air. (Kodama et al., 1993; Jia et al., 2006).

The performance of the solid desiccant honeycomb rotary wheel depends, to a large extent, upon the characteristics of the desiccant materials such as high dehumidification capacity, fast kinetics, high stability, and the ability to regenerate at a low temperature. Silica gel is the most popular solid desiccant usually used in the honeycomb rotary wheel due to its low cost, high porosity, and excellent stability although it has a limited dehumidification capacity of 20%–30% of its initial weight and higher regeneration temperature of 60–100°C (Kuma and Hirose, 1996; La et al., 2010; Lee and Lee, 2012; Li et al., 2016). As we know, desiccant dehumidifies in that desiccants have open empty spaces between the molecules called capillaries. As such, the hygroscopic performance of the silica gel is determined by its intrinsic physical/chemical properties and the surface porous (capillary) structure including surface area, pore volume, average pore size, and pore size distribution, which are affected by the preparation procedures/variables of silica gel (Kodama et al., 1993; Neti and Wolfe, 2000; Liu, 2003; Manjunatha et al., 2013).

The general manufacturing variables of silica gel are quite complex including the type of starting materials, catalyst, solvent, reaction temperature, gelation schemes, drying temperature, etc. Many research works have investigated in detail the effects of the preparation procedure on the surface characteristics of silica gel. Especially for the silica gel used in the honeycomb rotary wheel with glass fibers as substrate, water glass is generally employed to act as the type of starting material due to its availability, low cost, and environment friendliness (Sarawade et al., 2010; O’Connor et al., 2016; Prasad et al., 2019). The different preparation procedures of silica gel by neutralization using different acids such as HCl, H_3_PO_4_, H_2_SO_4_, HNO_3,_ and HCOOH were investigated to obtain the different surface characteristics of silica gel and thus the different dehumidification properties, which are also adopted to manufacture industrially silica gel desiccant in the honeycomb rotary wheel (Zhang et al., 2003; White et al., 2011; Wu et al., 2018; Yang et al., 2020). However, the usages of strong acids are unfavorable to operation and the environment due to their corrosion or volatility (Wang et al., 2013; Wei et al., 2013; Su et al., 2021). Herein, to our knowledge, it is the first time in this work to report that CO_2_ as nontoxic greenhouse gas was employed to neutralize the starting material water glass to prepare silica gel desiccant in the honeycomb rotary wheel and the preparation variables including CO_2_ pressure and the reaction time were optimized, the resulting silica gel desiccant in the honeycomb rotary wheel exhibits excellent surface characteristics and thus good dehumidification characteristics.

## Experimental

### Preparation of honeycomb silica gel

The flat glass fiber paper (Ahlstrom-Munksjo glassfibre Oy, Kotka, Finland) was cut to be 10 × 10 cm square and was rolled down to be corrugated glass fiber paper with colloidal silica (10 wt%, Chemical Engineering Sci.and Tech. Ltd. Company, Henan, China). Then, the flat and the prepared corrugated glass fiber paper were stacked alternately with a thickness of 10 cm to form a honeycomb monolith. The as-obtained honeycomb monolith was impregnated with water glass (ca. 3.3 modulus, 40 wt%, Haobo Chemical Engineering Ltd. Company, Shandong, China) for 2 hours and taken out to dry incompletely. The water glass-impregnated honeycomb monolith was placed in a high-pressure reactor and subjected to CO_2_ at different pressure for 3–5 h. After the reaction was complete, the honeycomb monolith was washed to remove the carbonate until the solution is close to being neutral followed by the aging in the deionized water for 2 h at 40°C. Finally, the honeycomb monolith with silica gel was completed by drying and calcination.

### Characterization of honeycomb silica gel

The powder X-ray diffraction (XRD) patterns were collected on a Japan Shimadzu DX-7000 advanced X-ray diffractometer using the Cu Kα radiation with *λ* = 0.15418 nm over 2θ degree from 10° to 70° at a scan rate of 4° min^−1^. Scanning electron microscopy (SEM) images were performed on a JEOL JSM-6700M scanning electron microscopy. Nitrogen physisorption was carried out at −196 ℃ on a Micromeritics BeishiDe PS2-1529-B specific surface area and pore size analyzer. The Brunauer–Emmett–Teller (BET) surface area, pore volume, and pore size distribution were obtained. The samples were pretreated at 150 ℃ under a high vacuum for 12 h prior to the nitrogen adsorption-desorption test. The pore volume was calculated from the adsorbed nitrogen after complete pore condensation at the relative pressure of P/P_0_ = 0.995. The pore size was estimated from the desorption branch *via* the Barrett–Joyner–Halenda (BJH) method.

### Adsorption/desorption kinetics and isotherms of water vapor

The adsorption/desorption kinetics and isotherms of the water vapor on the silica gel were measured on a gravity dynamic vapor system (BSD-VVS, BeiShiDe Instrument). Before the measurement of vapor adsorption, the samples were dried at 200 ℃ for 2 h. Nitrogen was used as the dry carrier gas and the concentration of vapor was precisely controlled with a mass flow controller and real-time vapor concentration monitor. The sample weight change during water vapor sorption/desorption can be measured by an ultra-microbalance module. With the mass change to < 0.002 mass%/min, the samples were considered to reach the kinetic equilibrium. Based on the equilibrium moisture content at different relative humidity, water vapor isotherms can be plotted. The adsorption kinetics was obtained by measuring the weight change of the samples with time at 25 ℃ at different relative humidity (RH). The adsorption isotherms were plotted based on the equilibrium adsorption capacity at various RHS. The desorption kinetics was obtained by measuring the weight change of the samples with time at 87 ℃ . Regeneration of silica gel in multiple adsorption-desorption cycles was also obtained in the system.

## Results and discussion

**FIGURE 1:**
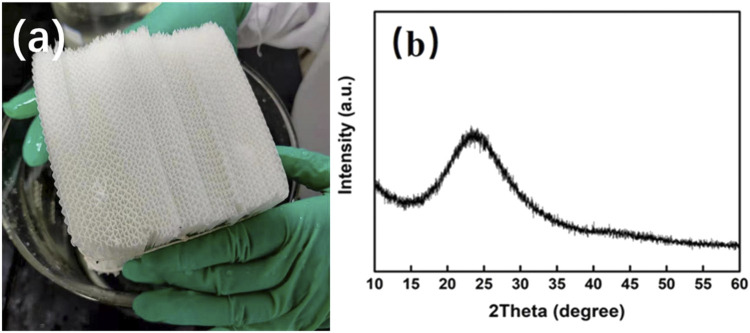
**(A)** Typical photograph of the as-obtained honeycomb silica gel with CO_2_ as the neutralized agent; **(B)** typical XRD pattern of silica gel powder detached from the honeycomb monolith.

Silica gel as a solid desiccant in a honeycomb rotary wheel is thought to be environmentally friendly and its desiccant capacity is dependent on its surface structure including specific surface area, pore volume, pore size, etc. As such, it is vital to choose a suitable and clean fabrication procedure to tune the surface structure of the silica gel solid desiccant in a honeycomb rotary wheel. Indeed, the surface structure of silica gel is affected by many preparation variables consisting of starting materials, catalyst, reaction temperature and time, aging temperature and time, etc. So, it is difficult to control elaborately the preparation procedure to obtain the desirable silica gel with excellent desiccant capacity (Zhang et al., 2006; Zheng et al., 2014). In this work, water glass was used as a green and low-cost raw material for the preparation of silica gel desiccant in a honeycomb rotary by the neutralization of acids. Although it is more difficult to result in the good surface structure of silica gel than organic silicon as a starting material because inorganic salt yielded during the neutralization in the solution impacts greatly the gelatinization of the silica sol, water glass could be used as an adhesive agent for the formation of honeycomb monolith without any other organic adhesive introduction. More importantly, CO_2_ gas as the neutralized acid should be able to relieve the salt effect in the solution due to the usage of liquid acid on the gelation of silica sol. As demonstrated in Figure 1 for the monolith and the XRD pattern of the as-obtained honeycomb silica gel by the reaction of water glass and CO_2_, it can be seen that there is a weak broadening band at about 23^o^ without any diffraction peaks of impurity, revealing the typical amorphous nature of SiO_2_ and thus a feasible preparation approach.

**FIGURE 2:**
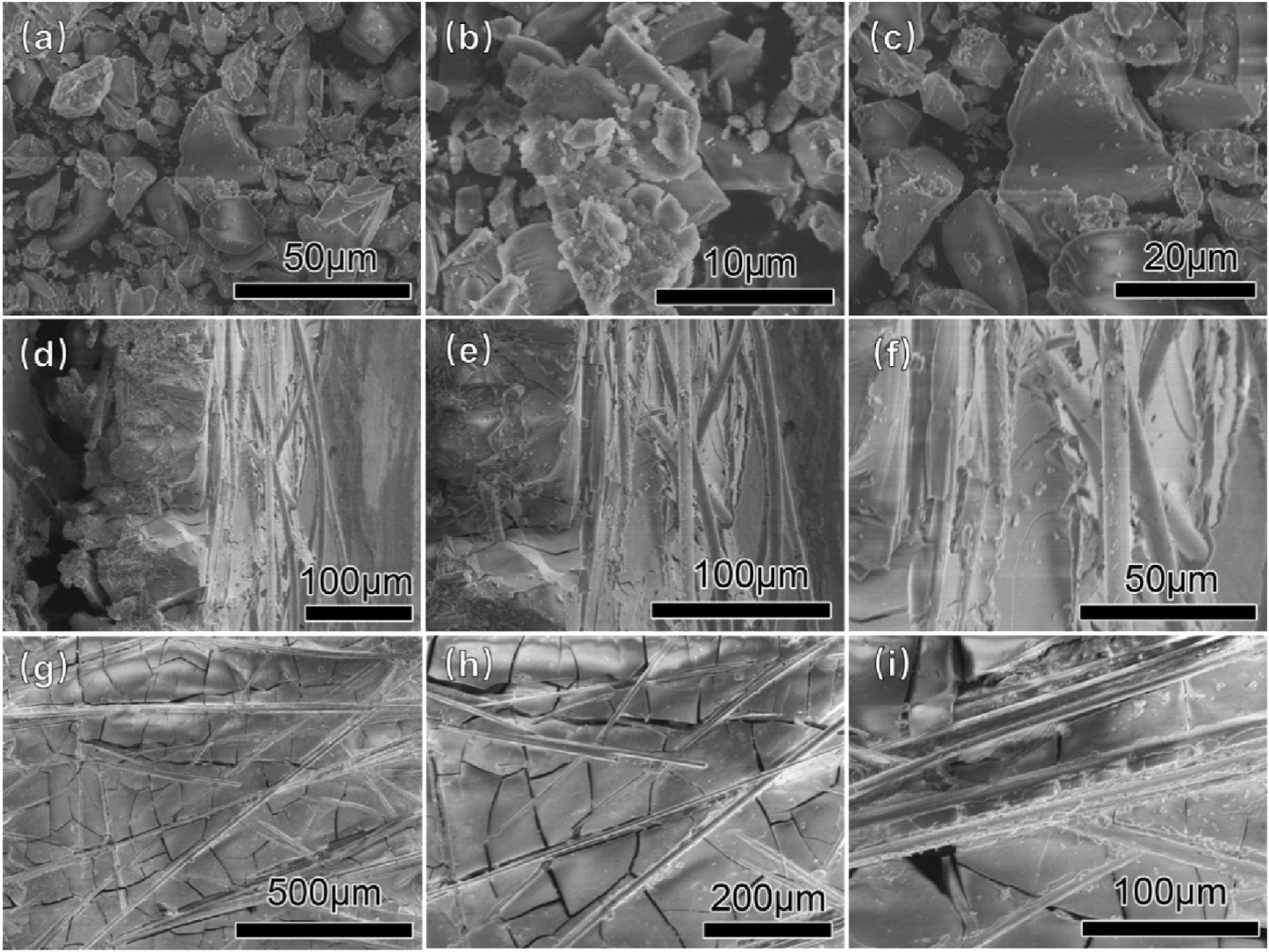
Typical SEM images of the as-obtained honeycomb silica gel with CO_2_ as the neutralized agent. **(A–C)** Silica gel powder detached from the honeycomb matrix; **(D–F)** cross section of honeycomb matrix; and **(G–I)** surface of the honeycomb matrix.

Figure 2 shows the SEM images of the obtained honeycomb silica gel at a different state. From Figures 2A–C, it can be seen that the prepared silica gel is gelatinized well with a smooth and uniform surface, which could be further verified by the SEM images of the cross section of honeycomb silica gel monolith in Figures 2D–F, illustrating the complete reaction of water glass with CO_2_ to implement the polymerization between SiO_2_ molecules during gelatinization. From Figures 2G–I, it is indicated that the prepared silica gel is dispersed evenly between the networks of glass fiber paper, allowing the available maximal exposed surface, which is favorable to promote the adsorption capacity. Although it is not observed obviously the surface microstructure of the prepared silica gel by glass fiber paper as a substrate, the texture of the monolith is indicated to disperse widely the silica gel on fiber gel paper so that the pretty large contact area of the silica gel with vapor for desiccant is obtained, which shall be verified further by the following characterized surface structure including specific surface area, pore volume, pore diameters.

**FIGURE 3:**
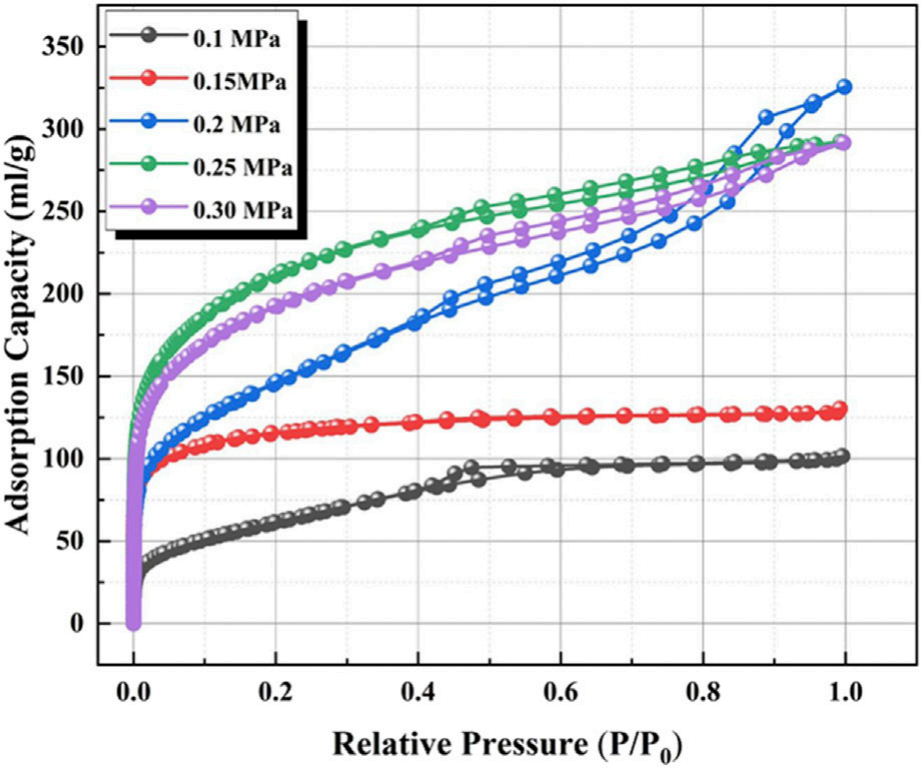
Nitrogen adsorption and desorption isotherms of the as-obtained honeycomb silica gel using water glass as the starting material at different CO_2_ pressures.

**FIGURE 4:**
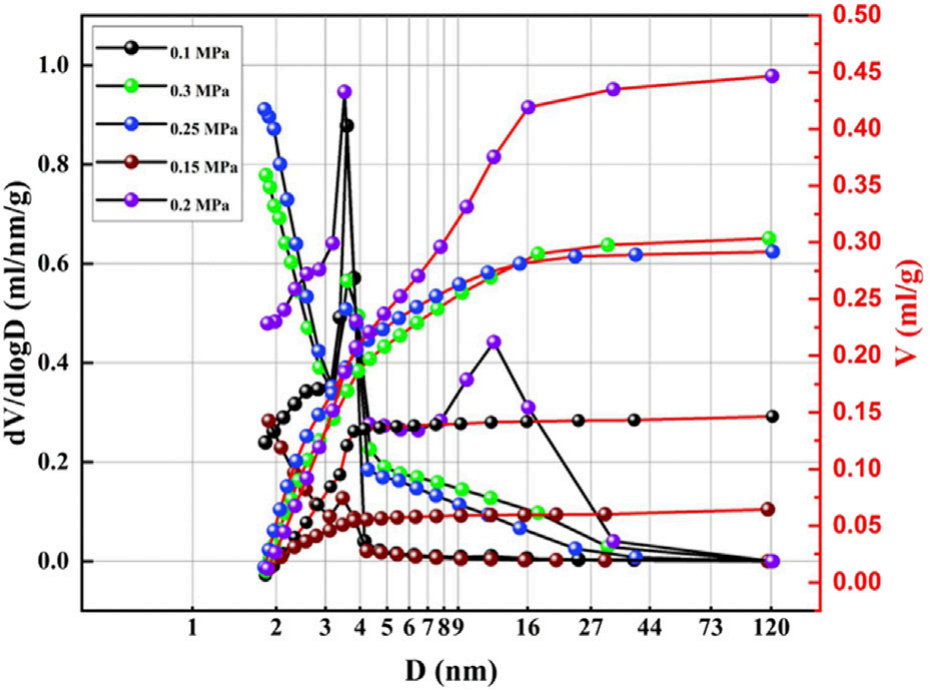
BJH (desorption) pore volume and pore size logarithm curves of the as-obtained honeycomb silica gel using water glass as the starting material at different CO_2_ pressures.

To optimize the effect of CO_2_ pressure on the surface structure, the preparation of the honeycomb silica gel using water glass as starting material was investigated at different CO_2_ pressure at ambient temperature. The nitrogen adsorption and desorption isotherms of the as-obtained honeycomb silica gel are shown in Figure 3. As indicated in Figure 3, all isotherms are identified as the typical Type IV, which are the characteristic isotherms of mesoporous materials at any pressure of CO_2_, preferably used as a desiccant as well. Nonetheless, the different isotherms at different pressure of CO_2_ demonstrate the different surface structures of the prepared honeycomb silica gel, displaying the regularity of CO_2_ on the surface structure of silica gel with the exception of the acid effect for the neutralization of water glass. As shown in Figure 4 for the changed surface structure with CO_2_ pressure including the pore size distribution, pore size, and pore volume, it could be seen that the most probable pore size between 2 and 10 nm is further evidence of the above typical Type IV BET isotherms of mesoporous materials although the pore size difference with CO_2_ pressure is markable, which should be embodied from the accumulative pore volume change with CO_2_ pressure. As known generally, the reaction of CO_2_ with water glass should be the neutralization of water glass by carbonic acid generated by CO_2_ and H_2_O, thus, CO_2_ pressure affects the reaction rate due to CO_2_ pressure dependence of CO_2_ concentration so that the surface structure of the generated silica gel is varied. All the detailed surface structure data of the prepared honeycomb silica gel at different CO_2_ were compiled in Table 1. From Table 1, it is clear that the surface structure of the prepared honeycomb silica gel is varied greatly with CO_2_ pressure, which could be explained by the famous Kelvin formula, as shown in the following equation:

$$RTln\frac{P_{2}}{P_{1}}=\frac{2\sigma M}{\rho }\left(\frac{1}{R_{2}}-\frac{1}{R_{1}}\right),$$ (1)

where *R* is the gas constant, *T* is the thermodynamic temperature of the system, *P*_*2*_ and *P*_*1*_ are the saturated vapor pressure at different curved diameters *R*_*2*_ and *R*_*1*_, respectively, *M* is the molar mass of water, *ρ* is the density of water.

**TABLE 1:** Surface parameters of the prepared silica gel at different CO_2_ pressures.

CO_2_ pressure (MPa)	Specific area (m^2^/g)	Pore volume (ml/g)	Average pore diameter (nm)
0.10	224.260	0.147	2.817
0.15	431.483	0.0644	2.563
0.20	523.207	0.292	4.088
0.25	764.862	0.447	2.938
0.30	695.765	0.303	3.316

From Eq. 1, it is clear that the saturated vapor pressure at constant temperature is dependent on the pore diameter, and the larger the pore diameter, the larger the saturated vapor pressure. Thus, the adsorption capacity of desiccant is directly related to the pore volume and the specific surface area of desiccant with the average pore diameter of the mesopore (2–50 nm). Thus, the optimized CO_2_ pressure for the preparation of the honeycomb silica gel is about 0.25 MPa, which might be contributed to the unique gas acid effect with rapid diffusion.

**FIGURE 5:**
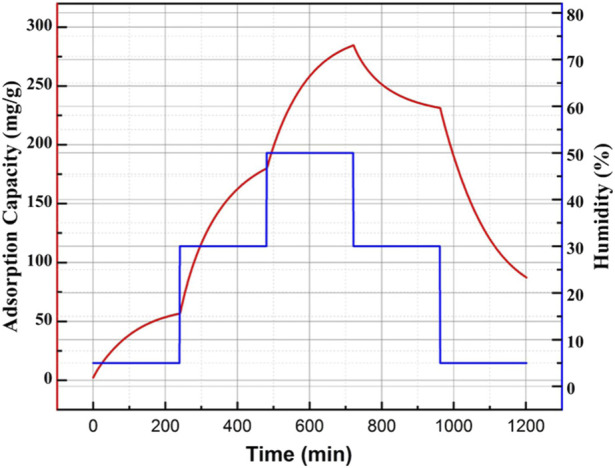
Typical adsorption/desorption kinetic curve at different humidities for the as-obtained honeycomb silica gel at a CO_2_ pressure of 0.25 MPa using water glass as the starting material.

To investigate the adsorptive characteristic of the prepared honeycomb silica gel, the adsorption/desorption kinetics was carried out at different humidity, as shown in Figure 5 for the typical adsorption/desorption kinetics at different humidity for the as-obtained honeycomb silica gel at CO_2_ pressure of 0.25 MPa. From Figure 5, it can be seen that the prepared silica gel at CO_2_ pressure of 0.25 MPa exhibits adsorptive ability at different relative humidity, which is indispensable for desiccant dehumidification used in industry due to the hash requirement of products to air humidity. Moreover, it could be further desorpted after saturated adsorption, exhibiting the cycling adsorption/desorption characteristics. The comparative adsorption capacity and time for saturated adsorption of different silica gels prepared at different CO_2_ pressure at a relative humidity of 50% were compiled in Table 2. In comparison, the silica gel prepared at CO_2_ pressure of 0.25 MPa shows the best high adsorption capacity and the suitable time for saturated adsorption at the relative humidity of 50%, which is attributed to the optimized surface structure, as shown above.

**TABLE 2:** Adsorption capacity and time for saturated adsorption of silica gel prepared at different CO_2_ pressures at a relative humidity of 50%

CO_2_ pressure (MPa)	Adsorption capacity (mg/g)	Time for saturated adsorption (min)
0.10	127.801	517.20
0.15	194.121	712.67
0.20	238.286	722.03
0.25	287.244	721.6
0.30	264.386	721.25

## Conclusion

The honeycomb silica gel in a solid desiccant rotary wheel is fabricated environmentally friendly using water glass as starting material and CO_2_ as neutralized acid. Water glass is used as an adhesive agent as well allowing the formation of the honeycomb monolith without any additional organic adhesive agent and guarantees a clean manufacturing procedure. CO_2_ could tune the surface structure of silica gel including the specific surface area, pore volume, and pore size due to its rapid diffusion and pressure. The results indicate that the prepared honeycomb silica gel has a large specific surface area of as much as 760 m^2^/g, a large pore volume of 0.45 ml/g, and suitable mesoporous size of 2.94 nm at a CO_2_ pressure of 0.25 MPa, which allows the excellent performance of the prepared honeycomb silica gel as a solid desiccant with an adsorption capacity of 287.24 mg/g at a relative humidity of 50%. Therefore, the proposed green preparation procedure of the honeycomb silica gel as a solid desiccant is feasible and promising in the manufacturing of honeycomb rotary wheels for dehumidification.

## Data Availability

The original contributions presented in the study are included in the article/supplementary material; further inquiries can be directed to the corresponding author.
